# Clinical implications for patients treated inappropriately for community-acquired pneumonia in the emergency department

**DOI:** 10.1186/1471-2334-14-61

**Published:** 2014-02-05

**Authors:** Scott T Micek, Adam Lang, Brian M Fuller, Nicholas B Hampton, Marin H Kollef

**Affiliations:** 1 St. Louis College of Pharmacy, 4588 Parkview Place, St. Louis, MO 63110-1088, USA; 2 Creighton University, 2500 California Plaza, Omaha, NE 68178, USA; 3 Washington University School of Medicine, 660 South Euclid Avenue, Campus Box 8072, St. Louis, MO 63110, USA; 4 BJC Learning Institute, 8300 Eager Road, Mail Stop 92-92-241, St. Louis, MO 63144, USA; 5 Division of Pulmonary and Critical Care Medicine, Washington University School of Medicine, 660 South Euclid Avenue, Campus Box 8052, St. Louis, MO 63110, USA

**Keywords:** Pneumonia, Antibiotics, Resistant pathogens, Outcomes

## Abstract

**Background:**

Community-acquired pneumonia (CAP) is one of the most common infections presenting to the emergency department (ED). Increasingly, antibiotic resistant bacteria have been identified as causative pathogens in patients treated for CAP, especially in patients with healthcare exposure risk factors.

**Methods:**

We retrospectively identified adult subjects treated for CAP in the ED requiring hospital admission (January 2003-December 2011). Inappropriate antibiotic treatment, defined as an antibiotic regimen that lacked *in vitro* activity against the isolated pathogen, served as the primary end point. Information regarding demographics, severity of illness, comorbidities, and antibiotic treatment was recorded. Logistic regression was used to determine factors independently associated with inappropriate treatment.

**Results:**

The initial cohort included 259 patients, 72 (27.8%) receiving inappropriate antibiotic treatment. There was no difference in hospital mortality between patients receiving inappropriate and appropriate treatment (8.3% vs. 7.0%; p = 0.702). Hospital length of stay (10.3 ± 12.0 days vs. 7.0 ± 8.9 days; p = 0.017) and 30-day readmission (23.6% vs. 12.3%; p = 0.024) were greater among patients receiving inappropriate treatment. Three variables were independently associated with inappropriate treatment: admission from long-term care (AOR, 9.05; 95% CI, 3.93-20.84), antibiotic exposure in the previous 30 days (AOR, 1.85; 95% CI, 1.35-2.52), and chronic obstructive pulmonary disease (AOR, 2.05; 95% CI, 1.52-2.78).

**Conclusion:**

Inappropriate antibiotic treatment of presumed CAP in the ED negatively impacts patient outcome and readmission rate. Knowledge of risk factors associated with inappropriate antibiotic treatment of presumed CAP could advance the management of patients with pneumonia presenting to the ED and potentially improve patient outcomes.

## Background

Community-acquired pneumonia (CAP) is an important infection globally, and accounts for significant morbidity, mortality, and economic burden [1-5]. Mortality may be as high as 14% overall, whereas for the elderly, mortality reaches greater than 50% within 5 years [1,5]. Treatment of CAP is potentially compromised by the rapid emergence of bacterial resistance to the most commonly prescribed antibiotics, including beta-lactams, macrolides, and fluoroquinolones [6]. Even multidrug-resistant (MDR) strains (commonly defined as resistance to three or more of the commonly used classes of agents) are emerging [6]. Traditionally, pneumonia developing in recently hospitalized patients or those residing in long-term care facilities, nursing homes, or undergoing dialysis, has been classified and treated as CAP. However, a new pneumonia classification, distinct from CAP —healthcare-associated pneumonia (HCAP)—has been introduced to address patients outside of the hospital at risk of infection with MDR pathogens [7]. The 2005 American Thoracic Society/Infectious Disease Society of America (ATS/IDSA) guidelines also recognized HCAP as a new category of pneumonia [8].

Patients with HCAP risk factors are at increased risk of infection with MDR pathogens [9-16]. Physicians should be aware that microbiological features and clinical outcomes of patients with HCAP are more similar to patients with hospital-acquired pneumonia (HAP) than those with traditional CAP [9]. A critical disparity appears to be the more frequent administration of inappropriate antibiotic treatment in patients with HCAP compared to those with CAP, secondary to a higher incidence of antibiotic-resistant pathogens in HCAP [13]. However, factors associated with inappropriate antibiotic administration in the emergency department (ED), and the short- and long-term impact of inappropriate therapy has not been well studied. Therefore, we performed an investigation to assess the incidence of inappropriate initial antibiotic treatment of hospitalized patients with presumed CAP presenting to the ED. We also aimed to assess risk factors associated with inappropriate antibiotic treatment and its relationship to other clinical outcomes.

## Methods

### Study overview and subjects

We retrospectively identified all hospitalized adult (age >18 years) patients diagnosed and treated for culture-positive CAP in the ED between January 1, 2003 and December 31, 2011. Presumed CAP was defined as a new or progressive radiographic infiltrate plus one of the following: temperature greater than 38.3°C, WBC greater than 10,000/μL, or purulent tracheal secretions. One investigator (MHK), blinded to the clinical and microbiologic information adjudicated the chest imaging. To be included, patients had to be treated in the ED with a CAP antimicrobial regimen that included at our institution ceftriaxone plus azithromycin or moxifloxacin. Identification of pathogens was based on the results of cultures obtained in the ED from blood, pleural fluid, sputum, or the lower airways. A positive urinary antigen for *Legionella pneumophilia* serogroup 1 was used to document infection with this pathogen. The Washington University School of Medicine Human Studies Committee approved the study.

### Endpoints and covariates

Patients receiving inappropriate therapy, defined as an antibiotic regimen that lacked *in vitro* activity against the isolated pathogen, were compared to those receiving appropriate antibiotic therapy. Covariates of interest included patient demographics, severity of illness, the Charlson score, and antibiotic treatment. Demographic factors included age, gender, and race. Comorbidities potentially influencing the isolated pathogen included residence in a long-term care facility, hospitalization in the last 90 days, receipt of antimicrobials in the last 30 days, end-stage renal disease requiring hemodialysis, and immunosuppression. Immunosuppression was defined as the presence of the human immunodeficiency virus (HIV), active malignancy undergoing chemotherapy, or treatment with immunosuppressants (i.e., 10 mg prednisone or equivalent daily for at least 30 days or alternate agents). Disease severity was assessed with the CURB-65 score, along with the need for either intensive care unit (ICU) admission or mechanical ventilation [17]. Infection-related variables included the presence of bacteremia complicating the pneumonia, and the presence of polymicrobial infection. We classified the initial antibiotic regimen as appropriate if the antibiotic regimen demonstrated *in vitro* activity against the pathogen(s) isolated and was administered within 6 hours of presentation.

### Statistics

We completed univariate analyses with either the Fisher’s exact test or Student’s t-test as appropriate. Continuous, non-parametrically distributed data was compared via the Mann–Whitney U test. All analyses were two tailed, and a p value of < 0.05 was statistically significant. Logistic regression was used to determine independent factors associated with inappropriate treatment. Variables significant at p < 0.10 in univariate analyses were entered into the model. To arrive at the most parsimonious model we utilized a step-wise backward elimination approach. Co-linearity was explored with correlation matrices. Adjusted odds ratios (AORs) and ninety-five percent confidence intervals (CIs) are reported where appropriate. The model’s goodness-of-fit was assessed via calculation of the R2 value and the Hosmer-Lemeshow c-statistic. All analyses were performed with SPSS 11.0 (SPSS, Chicago, IL).

## Results

**Figure 1:**
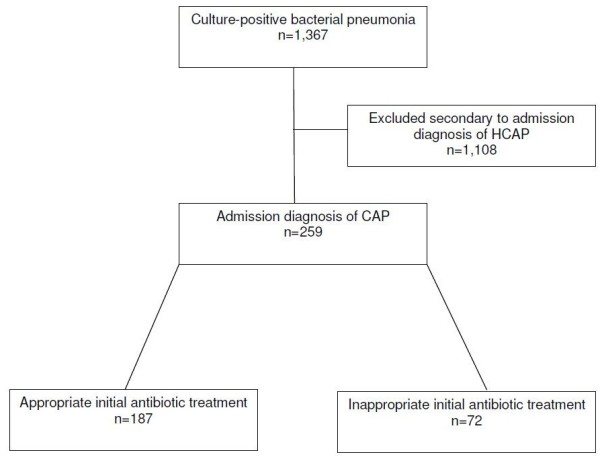
**Flow diagram depicting bacterial pneumonia patients admitted from the emergency department.** HCAP: healthcare-associated pneumonia; CAP: community-acquired pneumonia.

During the study period there were 1367 patients with culture-positive bacterial pneumonia admitted from the ED. Among these 1108 (81.5%) were diagnosed and treated for presumed HCAP, received initial treatment with broad-spectrum antibiotics and excluded from analysis. Of the 259 patients diagnosed and treated for CAP, 187 (72.2%) received appropriate antibiotic treatment and 72 (27.8%) received inappropriate treatment (Figure 1). Overall hospital mortality was 7.3% and 30-day hospital readmission was 15.4%. The average hospital length of stay was 7.9 days (median 5 days).

Table 1 shows the characteristics of patients receiving appropriate and inappropriate antibiotic treatment. There were no differences in demographics or severity of illness between groups. There was a trend towards greater ICU admission for patients receiving inappropriate treatment (p = 0.052).

**Table 1:** Baseline characteristics

**Variable**	**Appropriate initial therapy (n = 187)**	**Inappropriate initial therapy (n = 72)**	**P value**
Age, yrs:	58.1 ± 17.6	62.0 ± 15.3	0.103
Male, n (%):	109 (58.3)	40 (55.6)	0.690
Race, n (%):			
Caucasian	86 (46.0)	31 (43.1)	0.779
African-American	98 (52.4)	40 (55.6)	
Other	3 (1.6)	1 (1.4)	
CURB65	1.9 ± 1.4	2.1 ± 1.2	0.348
Charlson comorbidity score	4.5 ± 3.3	5.4 ± 1.2	0.090
Coexisting conditions, n (%):			
Coronary artery disease	34 (18.2)	14 (19.4)	0.815
Congestive heart failure	56 (29.9)	31 (43.1)	0.045
Peripheral vascular disease	13 (7.0)	13 (18.1)	0.008
Cerebral vascular accident	18 (9.6)	10 (13.9)	0.322
Dementia	4 (2.1)	0 (0.0)	0.578
Chronic obstructive pulmonary disease	93 (49.7)	48 (66.7)	0.014
Cirrhosis	23 (12.3)	6 (8.3)	0.364
Diabetes	57 (30.5)	24 (33.3)	0.657
Chronic kidney disease	20 (10.7)	16 (22.2)	0.016
Underlying malignancy	32 (17.1)	12 (16.7)	0.932
HIV positive	1 (0.5)	0 (0.0)	1.000
Healthcare-associated risk factors, n (%):			
Hospitalized in preceding 90 days	36 (19.3)	22 (30.6)	0.051
Long-term care resident	2 (1.1)	7 (9.7)	0.002
Chronic hemodialysis	3 (1.6)	6 (8.3)	0.016
Immunocompromised	38 (20.3)	17 (23.6)	0.562
Previous antibiotics	43 (23.0)	28 (38.9)	0.010
Number of healthcare risk factors:	0.7 ± 0.9	1.1 ± 1.0	<0.001
Healthcare-associated infection:	79 (42.2)	50 (69.4)	<0.001
ICU admission, n (%):	52 (27.8)	29 (40.3)	0.052
Respiratory failure, n (%):			
None	142 (75.9)	51 (70.8)	0.630
Intubation/mechanical ventilation	39 (20.9)	19 (26.4)	
Mask ventilation	6 (3.2)	2 (2.8)	
Hypotension, n (%):	110 (58.8)	45 (62.5)	0.589

Values are expressed as mean ± SD. CURB65 = confusion of new onset, urea greater than 7 mmol/l, respiratory rate of 30 breaths per minute or greater, blood pressure less than 90 mmHg systolic or diastolic blood pressure 60 mmHg or less, age 65 or older; HIV = human immunodeficiency virus; ICU = intensive care unit. The Charlson comorbidity score predicts the ten-year mortality for a patient who may have a range of comorbid conditions, such as heart disease, AIDS, or cancer (a total of 22 conditions).

With respect to infection-related characteristics, patients receiving appropriate treatment were more likely to be infected with *Streptococcus pneumoniae*, MSSA, *Escherichia coli* and *Haemophilus influenzae* (Table 2). MRSA, *Pseudomonas aeruginosa*, *Enterobacter* species and *Acinetobacter* species were more common among patients receiving inappropriate treatment. MRSA was the most common pathogen accounting for inappropriate treatment (47.2%) followed by *Pseudomonas aeruginosa* (27.8%). Tracheal aspirates and bronchoalveolar lavage specimens were more often used to establish the microbiologic diagnosis among patients receiving inappropriate treatment. There was no difference in the presence of polymicrobial infection or secondary bacteremia between groups.

**Table 2:** Infection characteristics

**Variable**	**Appropriate initial therapy (n = 187)**	**Inappropriate initial therapy (n = 72)**	**P value**
Cultures source, n(%):			
Blood only	46 (24.6)	13 (18.1)	0.041
BAL	5 (2.7)	6 (8.3)	
Sputum	105 (56.1)	34 (47.2)	
Tracheal aspirate	31 (16.6)	19 (26.4)	
*Achromobacter* species	0 (0.0)	1 (1.4)	0.278
*Acinetobacter* species	1 (0.5)	5 (6.9)	0.007
*Citrobacter* species	1 (0.5)	0 (0.0)	1.000
*Enterobacter* species	1 (0.5)	6 (8.3)	0.002
*Escherichia coli*	11 (5.9)	0 (0.0)	0.038
*Haemophilus influenzae*	36 (19.3)	3 (4.2)	0.002
*Klebsiella pneumoniae*	7 (3.7)	1 (1.4)	0.450
*Legionella* species	1 (0.5)	0 (0.0)	1.000
Methicillin-resistant *Staphylococcus aureus*	0 (0.0)	34 (47.2)	<0.001
Methicillin-susceptible *Staphylococcus aureus*	36 (19.3)	2 (2.8)	<0.001
*Moraxella catarrhalis*	5 (2.7)	0 (0.0)	0.326
*Proteus* species	1 (0.5)	1 (1.4)	0.479
*Pseudomonas aeruginosa*	0 (0.0)	20 (27.8)	<0.001
*Streptococcus pneumoniae*	79 (42.2)	3 (4.2)	<0.001
Other Streptococci species	22 (11.8)	3 (4.2)	0.097
Polymicrobial, n (%):	14 (7.5)	5 (6.9)	0.881
Positive blood culture, n (%):	51 (27.3)	17 (23.6)	0.549

*BAL* bronchoalveolar lavage.

We observed an association between comorbidities and inappropriate treatment. The median Charlson score was higher in patients receiving inappropriate treatment (5 vs. 4, p = 0.090). Neurologic conditions such as dementia and stroke occurred equally between groups while other chronic diseases, such as chronic obstructive pulmonary disease (COPD), chronic kidney disease, and peripheral vascular disease occurred more often in patients receiving inappropriate treatment. Of the comorbidities evaluated, those linked to the definition of HCAP correlated with inappropriate treatment (Table 1). For example, long-term care patients were more than nine times (OR, 9.96; 95% CI, 2.02-49.18) as likely to receive inappropriate treatment. Patients on chronic dialysis (OR, 5.58; 95% CI, 1.36-22.93) or having received previous antibiotic treatment (OR, 2.12; 95% CI, 1.18-3.79) were also significantly more likely to receive inappropriate treatment.

Overall, 129 (49.8%) patients were retrospectively found to have at least one HCAP risk factor. Patients having any of the HCAP risk factors were significantly less likely to receive appropriate antibiotic treatment compared to patients with no HCAP risk factors (61.2% versus 83.1%; p < 0.001). Inappropriate antibiotic treatment was not associated with excess mortality (Table 3). However, hospital length of stay was significantly longer among patients receiving inappropriate treatment. Similarly, 30-day hospital readmission was statistically greater among patients who initially received inappropriate treatment.

**Table 3:** Outcomes

**Outcome**	**Appropriate initial therapy (n = 187)**	**Inappropriate initial therapy (n = 72)**	**P value**
Hospital mortality, n (%):	13 (7.0)	6 (8.3)	0.702
Hospital length of stay, days*:	7.0 ± 8.9	10.3 ± 12.0	0.017
	4.0 [3.0,8.0]	6.95 [4.0,12.45]	
30-day hospital readmission, n(%):	23 (12.3)	17 (23.6)	0.024

*Data expressed as mean ± SD and median with 25^th^ and 75^th^ percentiles.

The results of the logistic regression analyses are shown in Tables 4 and 5. Admission from a long-term care facility, antibiotic exposure in the previous 30 days, and COPD were independently associated with the administration of inappropriate treatment. A similar analysis using 30-day hospital readmission as the dependent outcome identified three independent risk factors for this outcome: antibiotic exposure in the previous 30 days, presence of peripheral vascular disease and increasing CURB-65 scores.

**Table 4:** Multivariate analyses of independent risk factors for inappropriate initial antibiotic treatment*

**Variable**	**Adjusted odds ratio**	**95% CI**	**P value**
Long-term care resident	9.05	3.93 – 20.84	0.008
Antibiotic exposure in previous 30 days	1.85	1.35 – 2.52	0.049
COPD	2.05	1.52 – 2.78	0.018

*Other covariates not in the table had a p value >0.05 including previous hospitalization, mechanical ventilation or mask ventilation, CURB65 score, Charlson score, congestive heart failure, peripheral vascular disease, chronic kidney disease, hemodialysis, and ICU admission (Hosmer-Lemeshow goodness-of-fit test, p = 0.726). ICU = intensive care unit; COPD = chronic obstructive pulmonary disease, CURB65 = confusion of new onset, urea greater than 7 mmol/l, respiratory rate of 30 breaths per minute or greater, blood pressure less than 90 mmHg systolic or diastolic blood pressure 60 mmHg or less, age 65 or older.

**Table 5:** Multivariate analyses of independent risk factors for 30-day hospital readmission*

**Variable**	**Adjusted odds ratio**	**95% CI**	**P value**
Antibiotic exposure in previous 30 days	2.12	1.45 – 3.10	0.047
Peripheral vascular disease	3.64	2.27 – 5.84	0.006
CURB 65 (1-point increments)	1.46	1.27 – 1.68	0.007

*Other covariates not in the table had a p value >0.05 including inappropriate initial antibiotic treatment, age, chronic dialysis, intensive care unit admission, COPD, congestive heart failure, and the Charlson score (Hosmer-Lemeshow goodness-of-fit test, p = 0.431). ICU = intensive care unit; COPD = chronic obstructive pulmonary disease, CURB65 = confusion of new onset, urea greater than 7 mmol/l, respiratory rate of 30 breaths per minute or greater, blood pressure less than 90 mmHg systolic or diastolic blood pressure 60 mmHg or less, age 65 or older.

## Discussion

This retrospective analysis of patients diagnosed and treated for CAP in the ED reveals that inappropriate antibiotic treatment is common and primarily due to the presence of antibiotic-resistant pathogens including MRSA and non-lactose fermenting Gram-negative bacteria (NLFGNB). We found that almost one half of the patients treated for CAP in the ED had at least one risk factor for healthcare-associated infection. Healthcare-associated risk factors, including prior antibiotic exposure and long-term care residency, were found to be predictors for inappropriate antibiotic treatment. Our study is unique in showing that prior antibiotic exposure, presumably by predisposing to inappropriate antibiotic treatment, may also be associated with subsequent 30-day hospital readmission.

These data add to the growing evidence elucidating the importance of discriminating between patients with pneumonia having healthcare-associated risk factors, including prior antibiotic exposure, from those without such risk factors. In one of the largest studies examining the epidemiology of pneumonia, approximately one-third of all patients with pneumonia admitted to the hospital were due to HCAP [9]. Subsequent studies have also documented that HCAP represents between 30% and 60% of pneumonias requiring hospitalization in the US [11,14,18,19], and this trend is also observed in Asia and Europe [10,12,13,20]. Our study also confirms that MRSA and NLFGNB are common pathogens in patients presenting to the hospital with pneumonia having HCAP risk factors [9,13,14].

The importance of initial antibiotic therapy in patients admitted to the hospital with pneumonia is suggested by a recent medico-economic analysis conducted by Oster et al. [21]. These investigators attempted to identify risk factors for initial treatment failure in patients with CAP in non-ICU settings, and to characterize the association between initial treatment failure and length of stay, total hospital charges, and mortality. Treatment failure was associated with higher case fatality (8.5% vs. 3.3%), longer hospital stays (mean [SD], 10.1 [8.1] days vs. 4.9 [3.3] days), and higher total hospital charges ($37,602 [$71,876] vs. $14,371 [$21,633]) (all comparisons, p < 0.01). An important limitation of their study is that the role of inappropriate initial antibiotic treatment was not examined.

The findings from our study also confirm the recent observation made by Shorr et al. that patients with pneumonia and healthcare-associated risk factors are more likely to require 30-day hospital readmission compared to pneumonia patients without healthcare-associated risk factors [22]. Shorr and coworkers found that admission from a long-term care residency, prior antibiotic administration, recent hospitalization, and immunosuppression were independent predictors for 30-day readmission. However, our study is unique in focusing on patients diagnosed and treated for presumed CAP in the ED and linking 30-day hospital readmission to the administration of inappropriate antibiotic treatment, which in turn was independently associated with prior antibiotic exposure. Prior antibiotic exposure has been shown to be an important risk factor for the development of certain types of pneumonia to include drug-resistant *Streptococcus pneumoniae*[23], ventilator-associated pneumonia [24] and HCAP [13]. The common link among these types of pneumonia is infection due to antibiotic-resistant bacteria. The presence of a nexus between physician-prescribed antibiotic therapy (e.g., prior antibiotic exposure, inappropriate antibiotic treatment) and hospital readmission underscores that modifiable patient characteristics may play a role as key determinants of whether a hospitalized patient with pneumonia eventually requires readmission.

There are several important limitations of our study that should be noted. First, the study was performed at an academic medical center and the results may not be generalizable to other institutions, especially those with a lower incidence of MDR pathogens. The large proportion of pneumonia patients with HCAP risk factors receiving broad-spectrum antibiotic therapy at our institution underscores this point. However, our findings are in agreement with those from other investigators supporting their overall validity [9-16,20]. Second, we did not examine all aspects of medical care that may have influenced the prescription of inappropriate therapy and the outcomes of our patient cohort. For example, lack of physician experience or knowledge regarding the importance of broader empiric antibiotic coverage for patients with pneumonia and healthcare-associated risk factors could have contributed to our results [25]. Third, the retrospective nature of this study limits our ability to infer causality between the identified risk factors and inappropriate antibiotic therapy. Fourth, we did not identify the specific causes or indications for the 30-day readmissions. It is possible that the cause of readmission in many cases may not have been related to the prior episode of pneumonia or inappropriate treatment of the pneumonia. Our analysis found that the presence of peripheral vascular disease, greater CURB-65 scores, and prior antibiotic exposure predispose to readmission. These could all be considered to be markers of greater co-morbidities or severity of illness accounting for the readmissions.

Another important limitation of our study is that not all healthcare-associated risk factors carry the same importance. A US study found recent hospitalization, residency in a long-term care facility, chronic hemodialysis, and admission to the ICU to be the best predictors of pneumonia due to an antibiotic-resistant pathogen [26]. These findings are similar to those from a European study that identified prior hospitalization, residence in a long-term care setting, and chronic renal failure as predictors of antibiotic resistance in pneumonia [27]. However, a study from South Korea found prior antibiotic exposure and recent hospitalization to be independent predictors for antibiotic resistance [28]. Similarly, not all investigators have found antibiotic resistance and inappropriate therapy to be problematic in patients with pneumonia and healthcare-associated risk factors [29,30]. Several recent studies suggest adverse outcomes with broader spectrum therapy of pneumonia [31,32]. This has resulted in a call to avoid the use of the term HCAP and instead to refer to certain patients with pneumonia as being at increased risk for antibiotic-resistance due to their specific risk profile [33]. Moreover, it is important to acknowledge that our study only included culture-positive patients. Therefore, our data represent the more severe end of the CAP/HCAP spectrum. The use of empiric HCAP regimens do not appear to be justified in culture-negative patients with less severe disease [34].

## Conclusions

In conclusion we found that inappropriate therapy for patients diagnosed and treated for CAP in the ED setting is relatively common. Patients receiving inappropriate therapy were significantly more likely to have HCAP risk factors compared to patients receiving appropriate therapy, suggesting that the identification of these risk factors, or recognition of their clinical importance, was underappreciated in these individuals. The association of inappropriate therapy with longer hospital lengths of stay and greater 30-day hospital readmission suggests that efforts to reduce inappropriate therapy in the ED would be beneficial. Several investigations have applied multi-disciplinary approaches to the management of such patients demonstrating improvements in antibiotic prescription to include more targeted use of antimicrobial agents [35,36]. Future studies evaluating rapid microbiologic techniques, informatics systems for the timely identification of prior antibiotic exposure, and utility of new antimicrobial agents are needed to improve the treatment strategies for patients with pneumonia [37]. In the interim, clinician identification of risk factors for antimicrobial resistance should be emphasized in patients presenting to the hospital with pneumonia in order to optimize the balance between providing appropriate therapy and avoiding unnecessary antibiotic exposure [38].

## Abbreviations

AOR: Adjusted odds ratio; ATS/IDSA: American Thoracic Society/Infectious Disease Society of America; CAP: Community-acquired pneumonia; CI: Confidence interval; COPD: Chronic obstructive pulmonary disease; ED: Emergency department; HAP: Hospital-acquired pneumonia; HCAP: Healthcare-associated pneumonia; HIV: Human immunodeficiency virus; ICU: Intensive care unit; MDR: Multidrug-resistant; MRSA: Methicillin-resistant *Staphylococcus aureus*; MSSA: Methicillin-susceptible *Staphylococcus aureus*; NLFGNB: Non-lactose fermenting Gram-negative bacteria.

## Competing interests

Kollef has served as a consultant, speaker for, or received grant support from: Cubist, Hospria, Merck, and Cardeas.

Dr. Micek has received grant support from Cubist, Forest, Optimer, Merck, and Pfizer.

The remaining authors have no potential conflicts.

## Authors’ contributions

MHK, STM, NBH: had full access to all of the data in the study and take responsibility for the integrity of the data and the accuracy of the data analysis; contributed to the study conception and design, statistical analysis, drafting of the manuscript, and have given approval to the final version. AL: had full access to all of the data in the study and takes responsibility for the integrity of the data and the accuracy of the data analysis; contributed to the study conception and data base construction, and drafting of the manuscript and has given approval to the final version. BMF: had full access to all of the data in the study and takes responsibility for the integrity of the data and the accuracy of the data analysis; contributed to the study conception and design and drafting of the manuscript and has given approval to the final version. All authors read and approved the final manuscript.

## Pre-publication history

The pre-publication history for this paper can be accessed here:

http://www.biomedcentral.com/1471-2334/14/61/prepub
